# Supplementary material for: Interspecific comparison of gene expression profiles using machine learning
Source: PLoS Comput Biol. 2023 Jan 10;19(1):e1010743. doi: 10.1371/journal.pcbi.1010743 (PMC9879537; doi:10.1371/journal.pcbi.1010743)
Supplement: S1 Fig — (PDF) [file pcbi.1010743.s001.pdf]

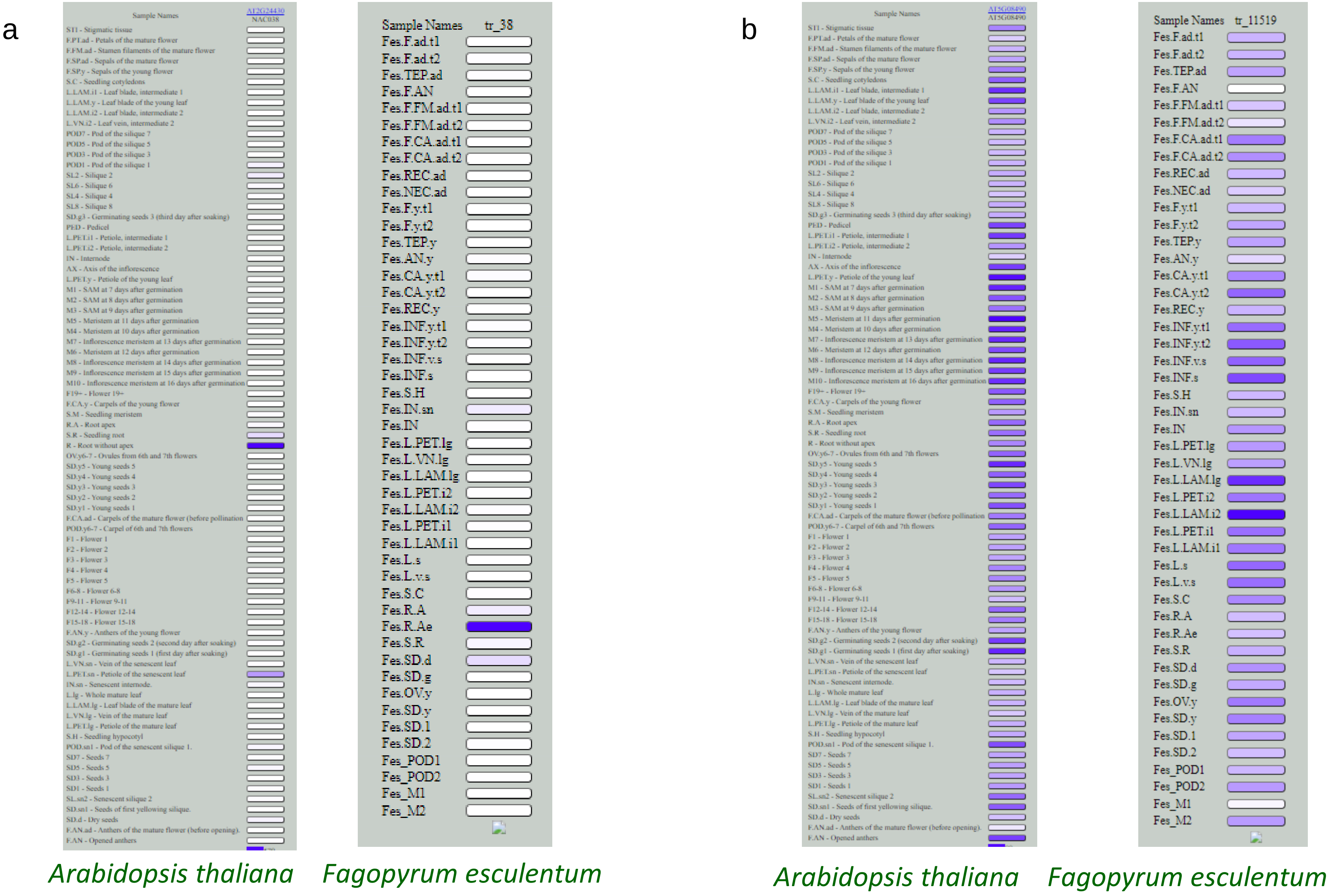

Figure S1. Example of the expression profiles in orthopairs. Color intensity denotes expression level. Panel **a** represents the orthopair AT2G24430 – tr_38, which has narrow expression pattern confined to roots, panel **b** – the orthopair AT5G08490 – tr_11519 which has wide expression pattern. The profiles are taken from the database TraVA, travadb.org
