# Supplementary figures and images for: Interspecific comparison of gene expression profiles using machine learning
Source: PLoS Comput Biol. 2023 Jan 10;19(1):e1010743. doi: 10.1371/journal.pcbi.1010743 (PMC9879537; doi:10.1371/journal.pcbi.1010743)

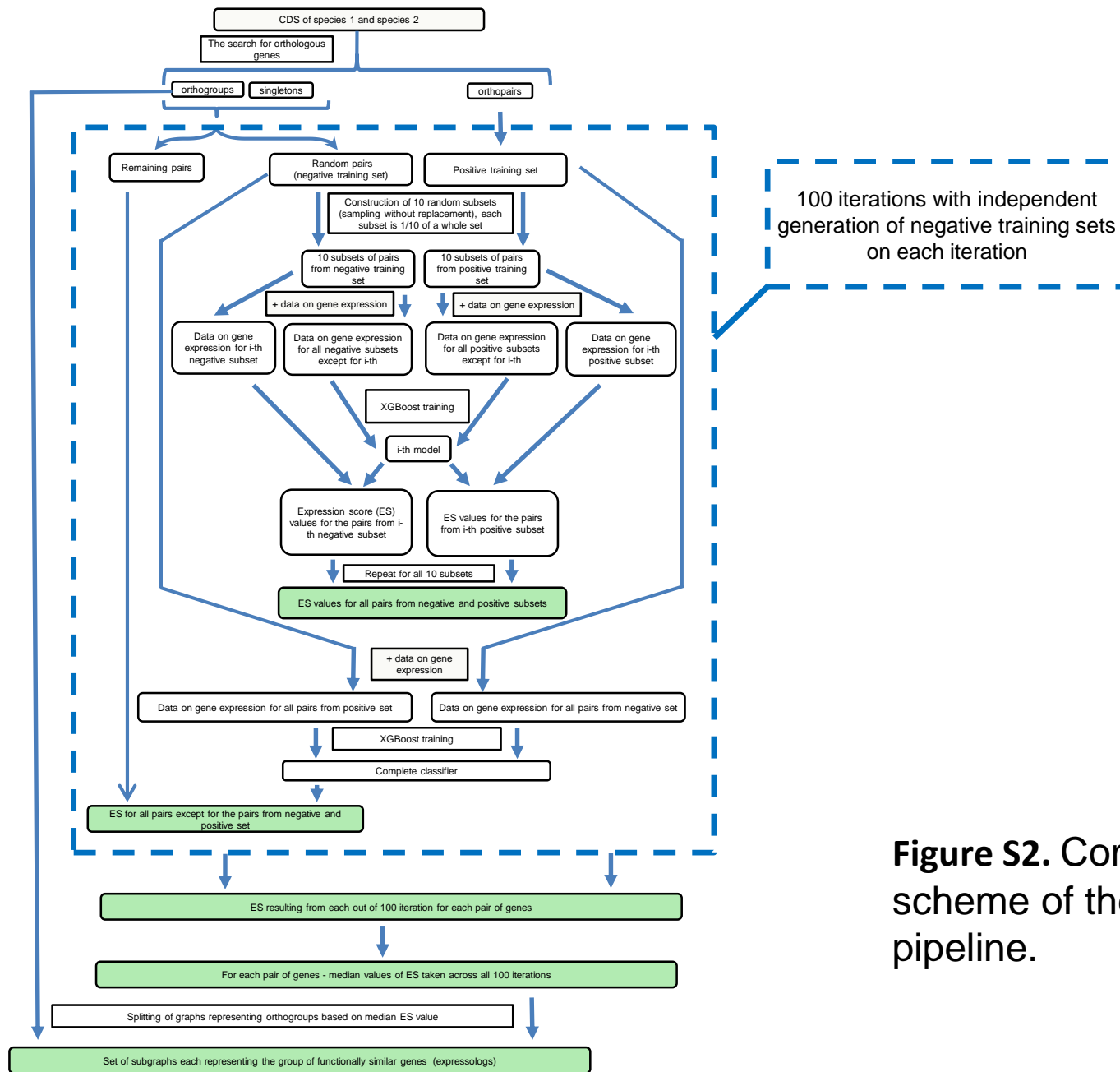

**Figure S2.** Complete scheme of the ISEEML pipeline.

Supplement: S2 Fig — (PDF) [file pcbi.1010743.s002.pdf]

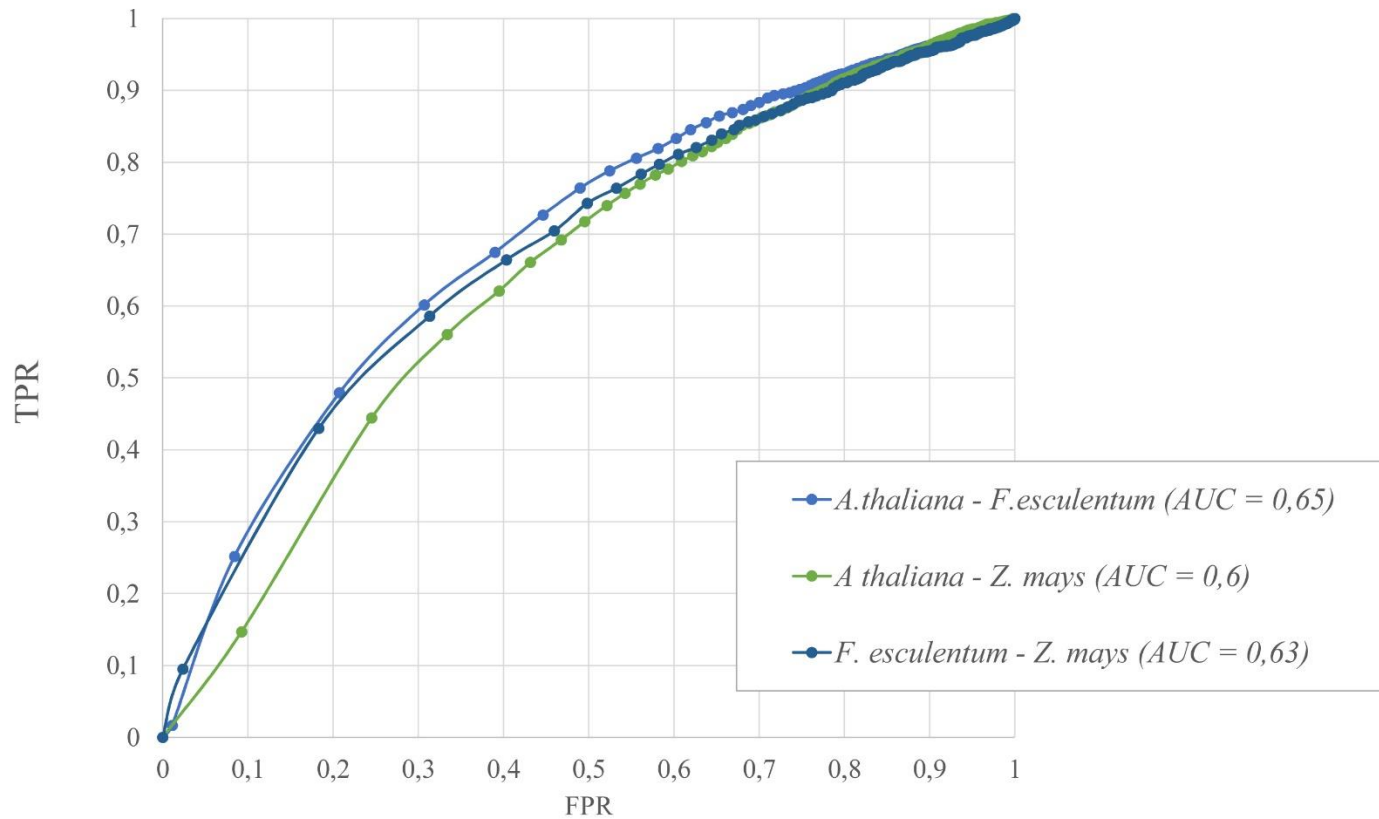

**Figure S10. ROC curves for the classifier based on Euclidean distance**

Supplement: S10 Fig — (PDF) [file pcbi.1010743.s010.pdf]
