# Supplementary material for: Interspecific comparison of gene expression profiles using machine learning
Source: PLoS Comput Biol. 2023 Jan 10;19(1):e1010743. doi: 10.1371/journal.pcbi.1010743 (PMC9879537; doi:10.1371/journal.pcbi.1010743)
Supplement: S3 Fig — Color intensity denotes expression level. (PDF) [file pcbi.1010743.s003.pdf]

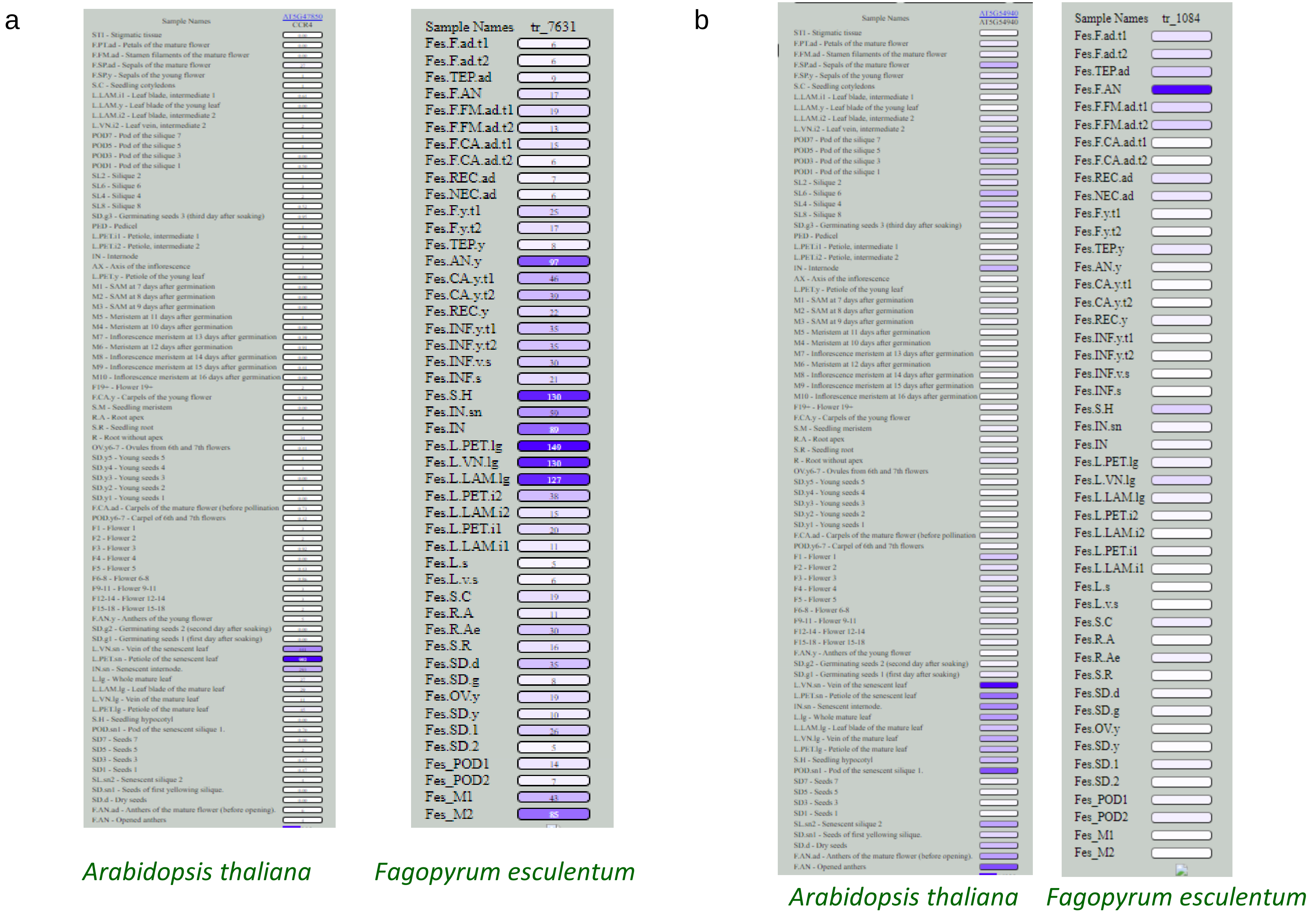

Figure S3. Example of the drastically different expression profiles in orthopairs. Color intensity denotes expression level. Panel **a** represents the orthopair AT5G47850 – tr_7631 (ES = 0.08), panel **b** – the orthopair AT5G54940 – tr_1084 (ES = 0.39). The profiles are taken from the database TraVA, travadb.org
