# Supplementary material for: Interspecific comparison of gene expression profiles using machine learning
Source: PLoS Comput Biol. 2023 Jan 10;19(1):e1010743. doi: 10.1371/journal.pcbi.1010743 (PMC9879537; doi:10.1371/journal.pcbi.1010743)
Supplement: S4 Fig — (PDF) [file pcbi.1010743.s004.pdf]

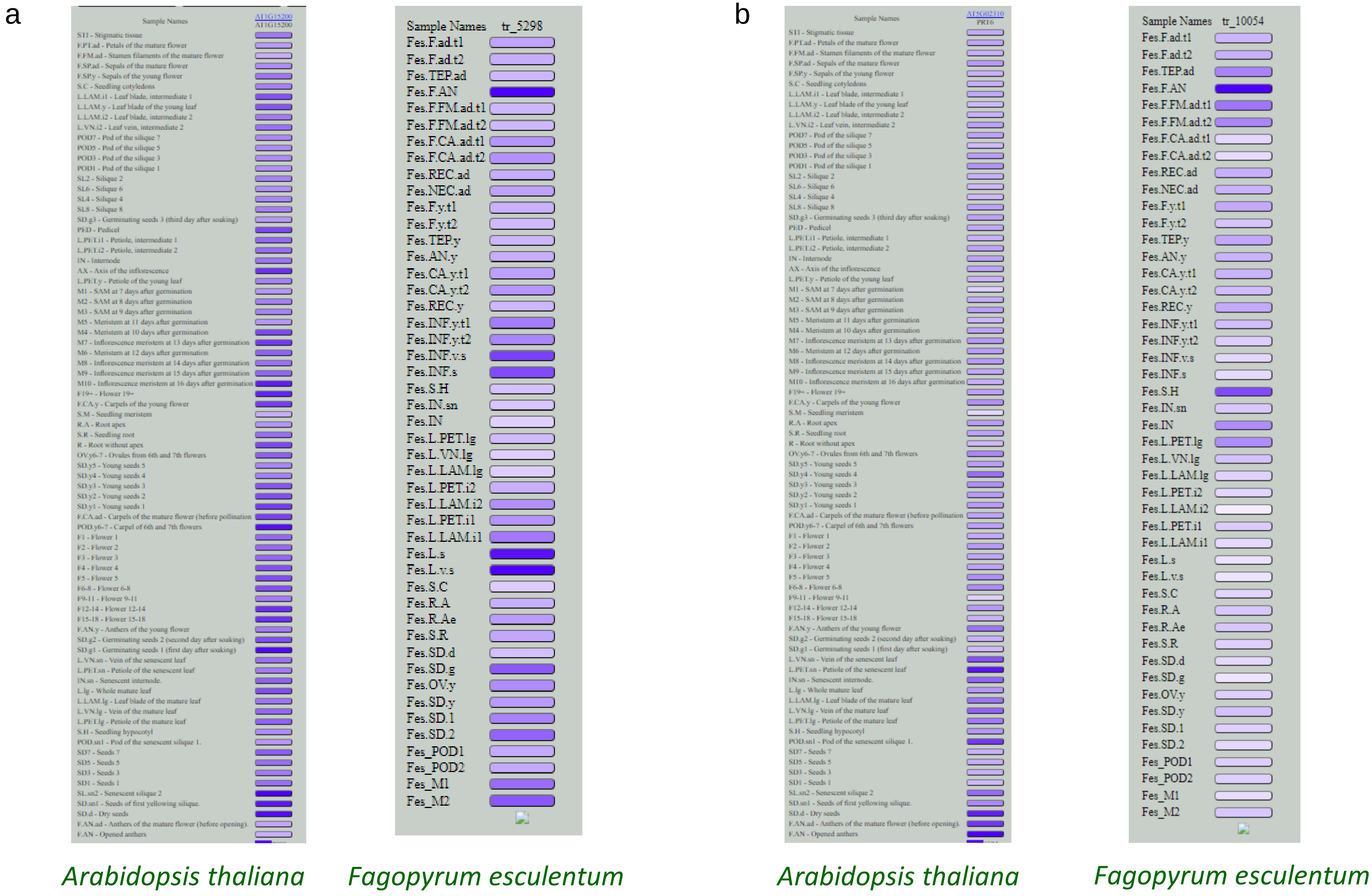

Figure S4. Example of expression profiles for gene pairs from the random pair set that have low identity but high ES. Panel **a** represents the pair AT1G15200 – tr_5298, panel **b** – the pair AT3G02310 – tr_10054. The profiles are taken from the database TraVA, travadb.org
