# Supplementary material for: Interspecific comparison of gene expression profiles using machine learning
Source: PLoS Comput Biol. 2023 Jan 10;19(1):e1010743. doi: 10.1371/journal.pcbi.1010743 (PMC9879537; doi:10.1371/journal.pcbi.1010743)
Supplement: S5 Fig — a. ROC curves for the classification of genes with narrow and broad expression patterns b. Precision-recall curves for the classification of genes with narrow and broad expression patterns (PDF) [file pcbi.1010743.s005.pdf]

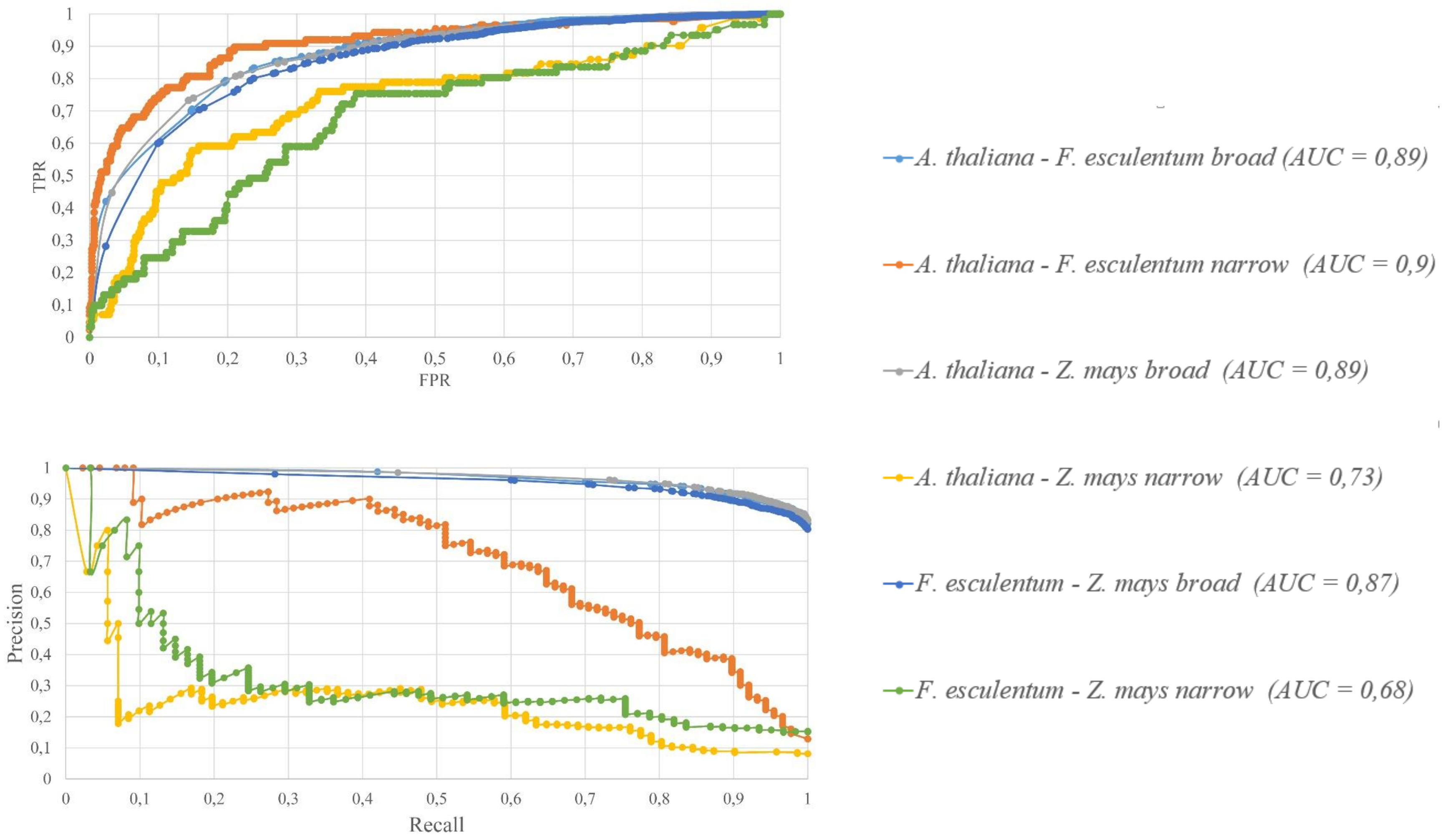


**Figure S5.** Classification of the genes with broad and narrow expression patterns. (A gene is considered as having narrow pattern if it is expressed in less than 10 samples for Arabidopsis and maize and 7 samples in buckwheat, broad pattern - if expressed in more than 70 samples for Arabidopsis, 55 for maize and 40 for buckwheat. The cut-off for the expression is 16 reads).
