# Supplementary material for: Interspecific comparison of gene expression profiles using machine learning
Source: PLoS Comput Biol. 2023 Jan 10;19(1):e1010743. doi: 10.1371/journal.pcbi.1010743 (PMC9879537; doi:10.1371/journal.pcbi.1010743)
Supplement: S8 Fig — (PDF) [file pcbi.1010743.s008.pdf]

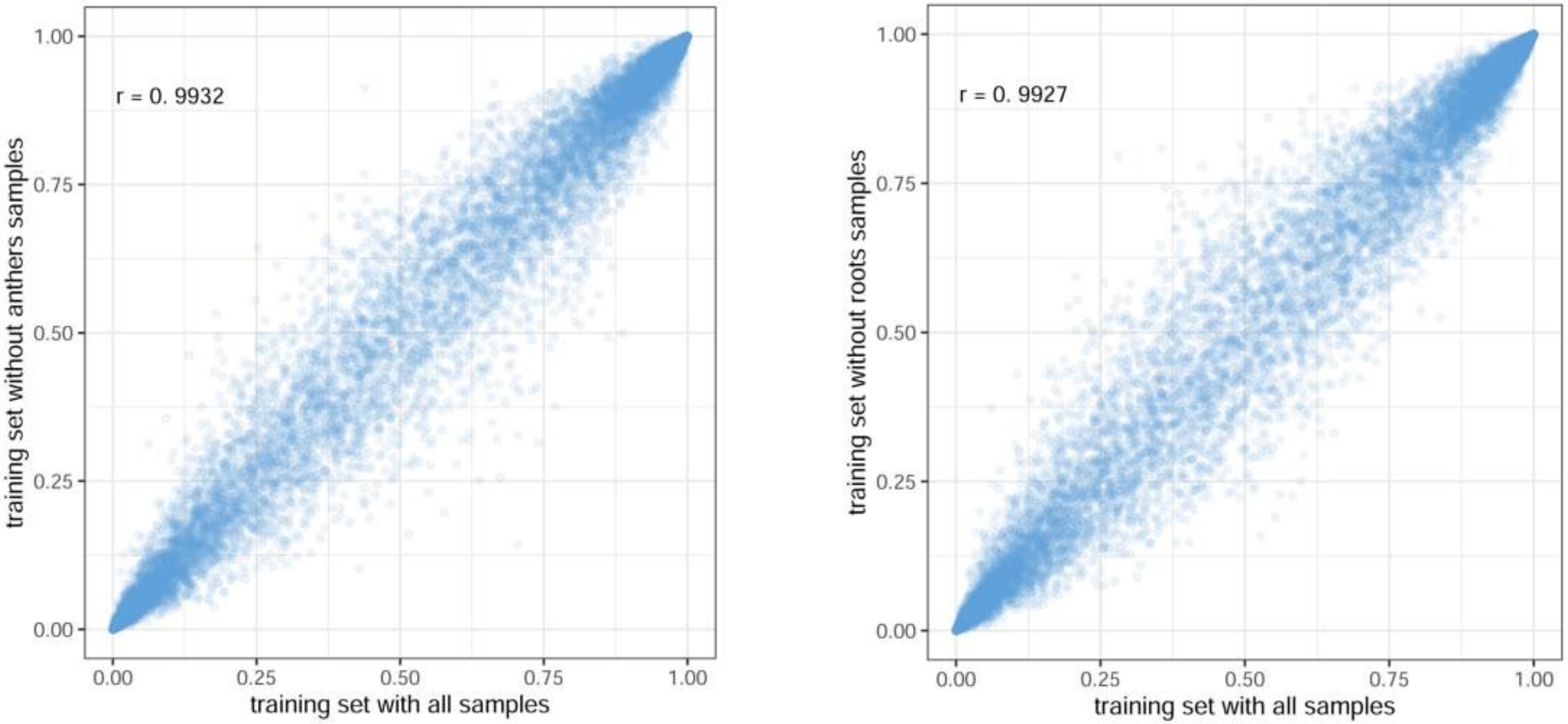

Figure S8. Comparison of the ES for orthopairs between ones inferred from the complete set and the set where some samples were removed (left – anthers excluded, right – root excluded).
