# Supplementary material for: Interspecific comparison of gene expression profiles using machine learning
Source: PLoS Comput Biol. 2023 Jan 10;19(1):e1010743. doi: 10.1371/journal.pcbi.1010743 (PMC9879537; doi:10.1371/journal.pcbi.1010743)
Supplement: S9 Fig — (PDF) [file pcbi.1010743.s009.pdf]

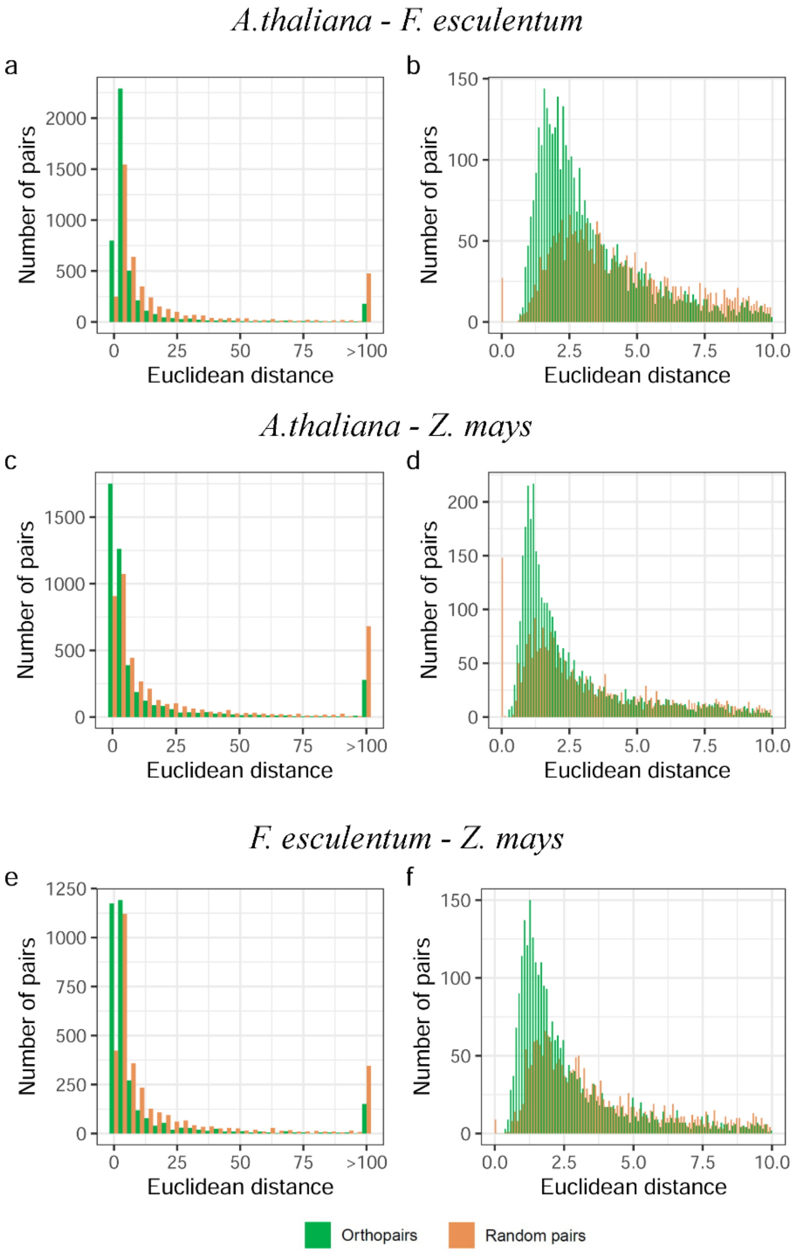

Figure S9. Distribution of Euclidean distances in orthopairs and random pairs (panels a, c and e show the complete range of values, b, d and f – the inset showing the distance in the range from 0 to 10.
