# Supplementary material for: Interspecific comparison of gene expression profiles using machine learning
Source: PLoS Comput Biol. 2023 Jan 10;19(1):e1010743. doi: 10.1371/journal.pcbi.1010743 (PMC9879537; doi:10.1371/journal.pcbi.1010743)
Supplement: S6 Fig — Big squares of orange, yellow, green, blue and violet color denote clusters. Samples in red squares—samples randomly selected from each cluster. (PDF) [file pcbi.1010743.s006.pdf]

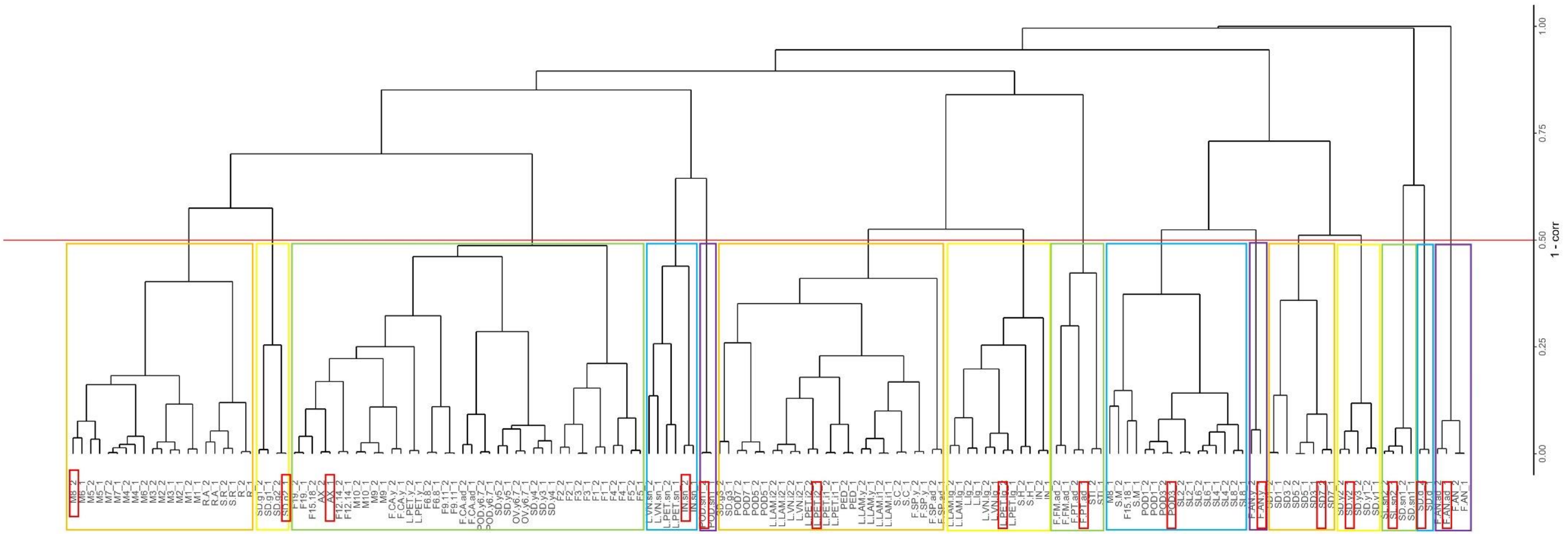

Figure S6. Example of the principle of sample selection in downsampling test. Horizontal red line denotes distance at which the tree was cut into clusters (0.5 in this example, varies from 0.1 to 0.9 in complete analysis). Big squares of orange, yellow, green, blue and violet color denote clusters. Samples in red squares - samples randomly selected from each cluster.
