# Supplementary material for: Interspecific comparison of gene expression profiles using machine learning
Source: PLoS Comput Biol. 2023 Jan 10;19(1):e1010743. doi: 10.1371/journal.pcbi.1010743 (PMC9879537; doi:10.1371/journal.pcbi.1010743)
Supplement: Attachment — Submitted filename: Response to Reviewers.pdf [file pcbi.1010743.s017.pdf]

Editor's letter

Dear Dr Penin,

Thank you very much for submitting your manuscript "Interspecies gene classifier based on the analysis of RNA-seq data using machine learning" for consideration at PLOS Computational Biology.

As with all papers reviewed by the journal, your manuscript was reviewed by members of the editorial board and by several independent reviewers. In light of the reviews (below this email), we would like to invite the resubmission of a significantly-revised version that takes into account the reviewers' comments.

We cannot make any decision about publication until we have seen the revised manuscript and your response to the reviewers' comments. Your revised manuscript is also likely to be sent to reviewers for further evaluation.

When you are ready to resubmit, please upload the following:

[1] A letter containing a detailed list of your responses to the review comments and a description of the changes you have made in the manuscript. Please note while forming your response, if your article is accepted, you may have the opportunity to make the peer review history publicly available. The record will include editor decision letters (with reviews) and your responses to reviewer comments. If eligible, we will contact you to opt in or out.

[2] Two versions of the revised manuscript: one with either highlights or tracked changes denoting where the text has been changed; the other a clean version (uploaded as the manuscript file).

Important additional instructions are given below your reviewer comments.

Please prepare and submit your revised manuscript within 60 days. If you anticipate any delay, please let us know the expected resubmission date by replying to this email. Please note that revised manuscripts received after the 60-day due date may require evaluation and peer review similar to newly submitted manuscripts.

Thank you again for your submission. We hope that our editorial process has been constructive so far, and we welcome your feedback at any time. Please don't hesitate to contact us if you have any questions or comments.

Sincerely, Andre Kahles, PhD Guest Editor PLOS Computational Biology

Ilya Ioshikhes Deputy Editor PLOS Computational Biology

Dear Editors,

thank you very much for the feedback and for giving us more time the revision. We throughly revised the manuscript and made all additional analyses requested by the reviewers, checked terminology and references etc. The detailed answers are given below.

One of the revisions required the involvment of a colleague who was not initially at the list of co-authors. So I inform that we included an additional co-author (Gleb S. Buzanov). All initial co-authors agree with this.

Reviewer 1

- Nice part

  Kasianov et al. describe a method to find what they call "expresso-groups" of genes and apply it to a pair of plant species. At the core is a tree-based binary classifier of gene pairs into expressologs and non-expressologs. It takes the vectors of unnormalized read counts in a number of libraries for each species as input and outputs an "ES" probability. The classifier is trained to reproduce orthologous pairs found with OrthoFinder. Their program ICML can predict orthology with reasonable accuracy using not sequence but expression profiles. I see a potential high relevance in complementing orthology search and for discovering interesting cases where the expression profile changed much but the sequence not or vice versa. Very importantly, the approach works on count matrices of shape g x n and G x m, where g and G are the numbers of genes in the two species, n and m are the numbers of RNA-Seq libraries from the two species and no meta information on the libraries is required (e.g. tissue types or conditions). The main difference to related previous work is that no correspondence between libraries is required and therefore n != m is allowed.

Answer to the Reviewer 1

We are grateful to the Reviewer for his/her valuable comments on our manuscript. We did our best to meet the Reviewer's requests. Below you can find our answers to the Reviewer's comments.

1) *The approach is genuine, generally applicable and of potentially broad interest. However, the manuscript requires more details and an improved readability. The differences between orthology and "expressology" have not been examined much and neither has the dependence on the library selection or evolutionary distance. The description of the ML method plays a minor role.*

   We performed additional analysis by running ICML procedure on two other pairs of species, which have greater evolutionary distance (150-130 m.y.a). The distant species is represented by Zea mays (maize), an agriculturally important plant for which extensive transcriptomic data are available. These data are derived from a diverse set of samples including ones grown in the field and is thus more prone to noise and biases

introduced by environmental conditions and technical factors. Despite that, the efficiency of classifier remains high at such distances and conditions.

We also perfomed the analysis based on downsampling - removal the part of the samples and showed it influence on the accuracy of estimation of expression scores.

Also we expanded the part on machine learning, in particular, added an alternative method employing neural networks.

2) *I found the title misleading as a gene classifier could for example find functional classes. Instead the authors present a way to learn the similarity of expression profiles of gene pairs.*

One of the main applications of the algorithm that we expect is the correspondence between genes of a model object, where function is known, and a non-model one, in order to find the gene (or genes) within the orthogroup which is the most likely to have the same function (approximated by the expression pattern). However we agree that in a current form the title could be non-specific and thus misleading. We will change it to more specific that mentions similarity of expression profiles but we would prefer to do this at later stages of the revision.

3) *"PLOS Computational Biology requires authors to make all author-generated code directly related to their study's findings publicly available" and "authors must clearly provide detail, data, code, and software to ensure readers' ability to reproduce the models". However, the ICML3 repository contains binary files (makemodel and predictAllByPortion) which appear to have the main functionality but it is not documented where they come from or for which platform they were compiled on. Also, several scripts mentioned in the manuscript are not in the repository, e.g. XGBoostTree.saveModel.py and PrintOrthogroupsAfterCutByTreshold.pl. Please make your source codes available. Please also state the program and version of XGboost you used and give a link to the repository*.

This is done

4) The method is not described carefully and thoroughly. It becomes only clear very late what the authors have done, e.g. they perform a binary classification of pairs of integer-valued vectors of sizes n and m. The description should be more formal, e.g. what kind of mathematical object is an expression map? Please mention early the size of n and m in your test data. Of what shape is the input to your classification trees?

We added this information in the manuscript.

5) In contrast to the protein sequences, the expression profiles are random (technical variation, biological variation, choice of tissue, age and conditions, no clear correspondence of samples) and may overall be insufficient to decide which genes are corresponding functionally, e.g. when the sampled libraries are not sufficient to discriminate between functions. High variation in the predicted orthology pairs could render the orthology-finding resuls largely irreproducible.

We tested the applicability of our approach based on alternative orthology inference method (Proteinortho). The results show that the efficiency of classification and its outcome are similar to that inferred from previously used method (Orthofinder).

6) A plausibility test in which the functional equivalence of all genes is known 100% would be to compare A. thaliana against itself. Only, the expression profiles would be calculated using different RNA-seq libraries. There are plenty of libraries is SRA to draw samples from. Further, a sample of a few pairs of species would give a better impression of the impact of the randomness that is additionaly introduced compared to a sequence based method.

We agree and we performed these additional calculations. We compared Arabidopsis genes against itself using two datasets - our transcriptome map (Klepikova et al. 2016, travadb.org) and several publicly available RNA-seq libraries. This resulted in a very high efficiency of classification (AUC = 0.99). Also, as described above, we run ICML pipeline for two additional pairs of species - Arabidopsis-maize and buckwheat-maize. For maize the data were obtained in totally different conditions and a different (though similar) library preparation protocol however the efficiency is still high.

7) ICML requires that one runs orthology-finding to obtain a training set. Moreover, if ICML achieved perfect accuracy it would render itself superfluous. It is therefore important to show that the disagreements between the ortho- and expressologs are not merely classification errors but have a biological meaning. This could for example be done for cases where one has a (partial) correspondence between libraries and can compare expression profiles with established methods.

We can not agree that it will be superfluous because ICML is not an alternative to orthology search, it is rather a means of the separation of co-orthologs into ones which are functionally similar (taking expression as proxy for function) and ones which are not. As for the incongruence between the orthology and expression score (the cases of orthologs which have low ES) we show several examples for the pair Arabidopsis-buckwheat where the genes are orthologs but their ES is low. In this pair the samples can be matched at least at the level of groups of samples (e.g. leaves, roots; floral organs) and one can see that the expression patterns are indeed very different. In particular, for Arabidopsis, the model object, there are many cases whether divergent expression pattern indicates on divergent function. This can be exemplified by the orthogroup “AT1G08260; AT2G27120; tr_14963” where two Arabidopsis co-orthologs arose due to lineage-specifc duplication. The ES in pair AT1G08260-tr_14963 is 0,9854 and in pair AT2G27120-tr_14963 it is 0,0185. Indeed, both AT1G08260 and tr_14963 have similar expression patterns, very broad ones, with maximal expression in leaves, meristems and inflorescences: http://travadb.org/browse/DeSeq/AT1G08260%20AT2G27120/RawNorm/AvNorm/Show=Yes/ (Arabidopsis, see profile for TIL1) and http://travadb.org/browse/Species=Fesc/tr_14963/RawNorm/Show=Yes/. This is congruent with the known function of AT1G08260 which is a catalytic subunit DNA polymerase epsilon and is thus expressed in every tissue which has dividing cells. The other co-ortholog, AT2G27120, has very narrow expression pattern mostly confined to anthers: http://travadb.org/browse/DeSeq/AT1G08260%20AT2G27120/RawNorm/AvNorm/Show=Ye

s/ (see profile for TIL2). This examples show the adequacy of the classification based on the ES and the association of the changes in expression patterns with the change of function.

8) In line 177, the authors imply that certain differences between ortho- and expresso-groups are due to neofunctionalization. This hypothesis is not supported by evidence. E.g.~it could be that a pair of samples from the two species can mostly be matched up well as they are from the same tissue, but one sample is perhaps under a certain condition that changes the expression level of a few genes only. A low ES score could merely be a misclassification error. Perhaps this error probability could be bounded with above plausibility test.

We agree that we can not distinguish between dramatic change of function (neofunctionalization) and the realization of similar function but in the other place/condition based on ES alone so we modified this sentence.

9) Can you please discuss why the large expresso-groups ("orthgroups") seem to include only 1 gene from one species and very many from the other species? It would suprise me if many gene families had several dozen genes of the same function in one plant but are single-copy in another plant. Could these be similarly regulated genes that have very different functions?

This is actually quite a typical situation for plants (and not only plants - lineage-specific expansions of gene families due to polyploidy and\or segmental duplications are found also in many invertebrates, fishes, amphibians - see e.g. https://bmcecolevol.biomedcentral.com/articles/10.1186/s12862-020-01650-3 , https://www.nature.com/articles/s41586-021-03198-8).

We added the information on that into the manuscript (in the introduction). Such genes often have similar functions and are redundant but in some cases, indeed, one or more members of the family can acquire new functions. The identification of the genes within a multigene family which have divergent expression patterns can help to reveal such cases - and this is actually one of the motivations for our study.

10) A discussion would be helpful on the influence you expect on the selection of samples, e.g. when you mainly have samples from xylem sap of one plant and mainly have samples from root from the other.

Since our approach is based on the classification of expression patterns there should be some overlapping diversity of samples. This is especially important for genes with narrow expression patterns (tissue- or organ-specific) because if they miss in one of the transcriptome maps they will not be efficiently classified. So the important requirement for the input samples is that they should represent a broad range of organs, tissues and stages. Such transcriptome maps are available for most model species in both animlas and plants and their number is growing for non-model as well.

11) Have the authors tried another ML method? Why have they chosen trees?

We also tried method based on neural networks and yielded similar results showing high efficiency of a classifier. We added the part describing the results.

- Minor Issues
    - typo in abstract: or -> of

      fixed

    - ES is used in the abstract before it is defined.

    - fixed

    - Line 120. I suggest to not use "classification" in that sense as readers of a paper with ML in the title will think of the usual meaning.

- we changed it to more specific term - “ inferred orthology”
    - Line 131. "set of expression levels" - is the *vector* of expression levels meant?

      Yes. We added this into the Methods section.

    - The caption of Figure 1 seems to be messed up as b) is a ROC curve, not a model.
    - We meant by the model the classifier under different training set. We changed the caption in order to make it clearer

    - Line 159. "identities" - state that this refers to a NW-Alignment.
    - this is fixed

    - In Figure 1e one cannot see how much the scatterplot is concentrated around the diagonal, as the dots overlap much.

- We have redrawn the plots and added correlation coefficient (please note that this part of figure is now moved to another part of the manuscript and to supplementary figures).
    - Line 185. oa -> of
- fixed
    - Grammar error in Figure 2a

      fixed

    - There is neither a caption nor a reference to Figure 2d.
    - fixed (we removed it because we do not discuss it)

    - Line 218. Why would the thresholding and collection of connected components not work on the basis of correlation coefficients in place of ES?

      Indeed, this is possible in principle but was not done in the previous studies that we cite in this sentence. We rephrased this.

    - Line 241. What is 'stream'?

    - we mean “thread”, just used wrong term, this is fixed now

- Line 246. Grammar error (plural singular mismatch).
- this is fixed
- Wrong indentation after line 284.
- this is fixed
- Give a reference for nwalign
- fixed
- Line 304: What are the weights you are referring to?
- The parameters in line 335 ff are not introduced.
- these are parameters of XGBoost, they are not specific to our method so we do not mentioned them in details.

- Line 165: From Figure 1b one cannot see the accuracy at a certain threshold. From Figure 1c) it looks like at the ES threshold of 0.5 the sensitivity should be >0.9 and the specificity is lower. Please give the formula and define what you refer to as specificity.

  we added the formulas

Reviewer 2

- Nice part

  The authors suggest a machine learning approach to refine orthology assignment for a pair of genes based on their expression pattern. The method assigns for each candidate pair an expression score. This score reflects the similarity of the expression pattern among the group of orthologous pairs compared to that of random pairs. The idea behind this method is very nice and shown to be potentially useful for refining orthology assignment, which is, traditionally, based on sequence identity.

Answer to the Reviewer 2

We are grateful to the Reviewer for his/her valuable comments on our manuscript. We did our best to meet the Reviewer's requests. Below you can find our answers to the Reviewer's comments.

1) My main concern is about the generality and applicability of the suggested method to other species. As the method is based solely on expression patterns, a major issue is how sensitive the results are to the quality of the data and its availability. There is a strong assumption that orthologous genes have similar expression patterns while it is unclear from this study how much it depends on:

a. The evolutionary distance of the compared species

b. The number of samples required to construct an informative and representative expression pattern

c. Should both species have expression patterns from similar tissues/organs? Same developmental stage? Similar environmental conditions?

We agree with this point and run several additional analyses aimed at answering these questions. In particular: 1) we added another species which has larger evolutionary distance with both buckwheat and arabidopsis 2) we performed a test showing the changes in the estimation of expression scores depending on the number of samples 3) we estimated the classification efficiency for the genes with broad and narrow expression patterns 4) we performed a self-constistency test based on a comparison of Arabidopsis genes against themselves using two different transcriptomic datasets (one is a map from Klepikova et al. 2016 and the other is a set of publicly available samples from SRA belonging to different studies).

2) I think that the sensitivity of the results should be rigorously tested also given these issues. For example, the authors show that the correlation scores with and without specific samples are still high (but do not quantify it). I would also like to see some analyses demonstrating, for example, how sensitive are the results to the number of samples used.

We made additional calculations that include the downsampling of a complete set and the comparison of expression scores resulting from such reduced dataset with ones based on complete set. We also added the correlation values for the results with specific samples removed.

3) In the current manuscript, the authors only state one sentence about this issue: "This finding shows that classification based on expression requires accurate sampling that reflects all organs and developmental stages." I think these analyses should be expanded. In addition, Could the authors evaluate their method also for different species with varying evolutionary distances?

We agree and expanded our analysis by incorporating another plant species for which a wealth of transcriptomic data is available - Zea mays (maize). Totally, analysis with three interspecific pairs was done: Arabidopsis - buckwheat, Arabidopsis-maize and buckwheat-maize. Maize belongs to monocots while Arabidopsis and buckwheat - to dicots. Monocot-dicot divergence is estimated to take place 130-150 m.y.a. The testing on more distant clades, e.g. gymnosperms vs flowering plants is not possible due to absence of high-resolution transcriptome data and very large genome with a suboptimal assembly and annotation. We did not perform tests for small evolutionary distances (e.g. Arabidopsis and other species from the same family, like Brassica) because in this cases previously existing approaches such as based on Euclidean distance or correlation work well enough, as shown in previous studies.

4) From a practical point of view, it would be nice if the authors could address how realistic the method is for non-model species. Could the authors suggest rules-of-thumb for the minimal requirements for the method to be applicable?

We anticipate that our approach is suitable for non-model species. The obvious limitation is the availability of the good annotation and the transcriptome map which is detailed enough to cover all critical samples. Though the annotation of eukaryotic genomes is still a challenging task there are a number of efficient pipelines (e.g. Braker2) for this. The availability of RNA-seq data, including high-resolution transcriptome maps, is also increasing due to the decrease of the costs and increase of throughput of sequencing. However, if a transcriptome map is not available for an object of interest, we recommend the following procedure: 1) to find a

transcriptome map for the model object closest to the object of interest 2) to characterize the similarity of the samples based on their expression profiles (this can be done, for example, by construction of a distance tree using “1 - correlation” as a measure of expression or by principal component analysis 3) to select the samples which represent all major clusters/groups 4) to collect them and do RNA-seq or to choose samples from publicly available in SRA/ENA.

5) Does the method explicitly account for the sequence similarity of the given candidate pair? It is not clear from the text, but it seems not? Would including this information as an additional feature improve the classification?

We do not directly use similarity values as a source of information for classification but since we perform classification within the orthogroups - the genes that by definition share common ancestor - they have certain similarity.

6) Concerning the previous point, I think a comparison of the suggested method with the classical reciprocal-best-hit (RBH) is missing. How much do we gain/lose from the new method compared to relying solely on sequence similarity or RBH.

In contrast to reciprocal-best-hit (RBH) approach we do not aim at the grouping of genes from different species according to their common descent (approximated by the sequence similarity) but at the grouping of genes according to their function (approximated by the expression patterns). We apply this procedure to the genes that were already assigned to a group sharing common descent (orthogroup) in order to find ones that have similar functions. Going back to RBH, it is not possible to use it for the inference of the orthogroups because it gives, by definition, only a pair of genes and is not well suitable for the groups with frequency of whole genome duplications (see e.g. https://www.ncbi.nlm.nih.gov/pmc/articles/PMC3814191/)

7) What is the correlation between the ES and the similarity/bit scores? I assume some of the FN (low ES scores) have high sequence similarity, is it just the 6% mentioned in the text?

The 6% mentioned in the text are the 1-to-1 orthologs with low ES. The identities in orthopairs (irrespective of ES) vary from 10% to 100% with maximum at 55-60%. The distributions for orthopairs with ES >0.5 and < 0.5 are given in the Figure 1d. It shows that these distributions greatly overlap though one can see a tendency for lower identities in the pairs with ES < 0.5.

8) As far as I understand, the method is designed to determine the 1:1 orthology relationship for a pair of candidate genes. Could it also be scaled to more than a pair of species? I think this would be critical for the current pan-genomic era. I think it should be further discussed.

Indeed, in its current implementation the pipeline does not support the multispecies analysis but we expect that this is feasible, at least for closely related species where a sufficient number of 1-to-1 orthogroups can be found. This is one of the directions of further development.

9) From a practical point of view, since the model needs to be trained specifically for the species under study, could the authors provide some general measures for the quality of the model? In addition, it is unclear how much the hyperparameters used for learning are applicable for a new dataset. More specifically: how many iterations should be used? What should be the ratio between the positive and negative set etc? Could this process be done automatically as part of the provided code? It currently seems that the method cannot be used out of the box with a new dataset. I'll be happy for some clarification and think a detailed protocol/tutorial could greatly enhance the applicability of the suggested method for other species.

We tested different values for several parameters and found that they have little influence of the efficiency of classifier (estimated by AUC). We found that the same parameters are optimal for different interspecific pairs thus we expect that they can be at least a good starting point for the analysis of new datasets. As for the automatic selection of parameters, actually the current version does not have this option.

- Minor

1. ES threshold is mentioned in the abstract but defined only much later in the text, please rephrase or better explain.

   we added a definition in the previois sentence

2. Could the authors better define and detail "a high-resolution transcriptome map".

   This is, of course, an debatable question ("How many is many?") but based on the survey of recent studies (e.g. transcriptome map of sheep: https://journals.plos.org/plosgenetics/article?id=10.1371/journal.pgen.1006997 , chicken: https://academic.oup.com/gigascience/article/7/7/giy086/5052204 , sorghum: https://onlinelibrary.wiley.com/doi/10.1111/tpj.13781) it ranges from 20 to 90 samples covering tissues and organs at different stages of development.

3. Are the AUC values reported based on the cross-validation average? Was part of the data used solely for training and another one for testing as commonly done in machine learning approaches?

We used a k-fold cross-validation. The negative and positive sets ere divided into 10 parts and then classifiers trained on 9 parts of the training sample were created, and then the remaining 1/10 part was used as a test sample. The procedure was repeated with each of the 10 parts.

4. I suggest the authors would use the precision-recall measure as it is less biased for unbalanced datasets (in this case ~4K positive to 1M negative) (https://www.biostat.wisc.edu/~page/rocpr.pdf)

   This is done.

5. What are the correlation values for Fig 1E?

   This is done (but this figure is moved to the supplement due to the high amount of new figures)

6. The authors suggest that 'true' orthopairs with low ES scores exemplify neofunctionalization events. It is not necessarily the case as it could reflect genes active in different environmental conditions / developmental stages etc. Accounting also for the sequence identity could potentially shade more light on this issue.

   We agree that we can not distinguish between dramatic change of function (neofunctionalization) and the realization of similar function but in the other place/condition based on ES alone so we modified this sentence.

7. What are the exact datasets used for this study? Which expression datasets were used for each species? Are they publicly available?

   Yes, all datasets are publicly available. We provided a list in the Supplementary tables S4 and S5.

8. Figure 2D legend is missing and not referred to from the main text

   indeed, we do not discuss and we removed it.

9. Why do the authors take the non-normalized read count as a feature? Wouldn't the normalized count remove some of the biases in each dataset? Does it matter?

   The methods of normalization which account for RNA composition (e.g. TMM or median of ratios used in Deseq2) can not be applied in this case because they require one-to-one correspondence between genes from different species which is not feasilble. Other methods, which account only for sequencing depth and gene length are by definition the multiplication on a constant - this procedure is not expected to change the outcome because we compare not absolute expression values but the the expression profiles which are based on the ratio of read counts in different samples.

10. Which version of OrthoFinder was used?

We added this information

Reviewer 3

- Nice part

In this manuscript, Kasianov et al. present a method to classify orthologs according to the similarity of their expression profiles. For this, they apply machine learning to expression counts and sequence similarity of genes which are already defined as orthologs (including many-to-many) between two species.

Answer to the Reviewer 3

We are grateful to the Reviewer for his/her valuable comments on our manuscript. We did our best to meet the Reviewer's requests. Below you can find our answers to the Reviewer's comments.

1) While the aim of the method is to discover genes with the same function, the concept of function is never defined in the context of this work.

We mean the function at the organismic level rather than the molecular function of a protein. We indicated this in the summary.

2) This manuscript takes as fact that expression is a good and sufficient proxy for function (although the ML does also use sequence similarity, this is never discussed), but there is only one reference given to support this, from a study which validated only 8 genes.

This is a usual and widespread assumption that underlies, implicitly or explicitly, most bioinformatics/systems biology studies dealing with gene expression. Despite it obviously has several limitations (mobile RNA, posttranscriptional and posttranslational regulation), this has been shown experimentally for several model objects (e.g. https://www.pnas.org/doi/10.1073/pnas.1415046112). In particular, for duplicated genes in mice it was shown that duplicated genes with similar expression pattern have lower probability to be essential (i.e. knockout of the gene generates lethal phenotype) than ones that have divergent expression patterns. This and many other experiments show that gene function and expression are tightly linked. We added these references and expanded this part of the text.

3) These two points affect all sentences with statements about function. Notably, lines 199-200 and 209, functional similarity or difference have not been shown.

We rephrased these sentences emphasizing the assumption of the association between function and expression pattern.

24) There are several statements comparing this method to others in principle, but it is very surprising for a methods paper to present no comparison to results from other methods. This is absolutely needed, with an exploration of sensitivity to potential confounding factors (e.g. genome annotation, evolutionary distance, quantity of RNA-seq data).

There is no direct analogs because this is, to our knowledge, the first study that uses machine learning for the evaluation of similarity of expression profiles between species. However the problem itself is not novel; in previous studies which compared expression maps in Drosophila and mammals it was tackled by using Euclidean distance for making the correspondence between samples (https://bmcecolevol.biomedcentral.com/articles/10.1186/s12862-015-0426-x , https://pubmed.ncbi.nlm.nih.gov/24101476/). We applied the same method for our data and made calculations for three pairs of species - Arabidopsis-buckwheat, Arabidopsis-maize and buckwheat-maize. The results show that a classifier based on Euclidean distance has very moderate success (AUC =0,65, 0,6 and 0,63 correspondingly). We included these results into the manuscript.

25) The authors cite as a shortcoming of other methods that they need to be applied to species similar enough to match samples, but the only application given is to two land plants. It would be more convincing to show that genes of similar function could be found between a plant and e.g. a yeast or a fly.

Since by the function we mean the function at organismal level so a certain level of similarity in the cellular organization, complexity and physiological reactions is required. Also, since the method is based on a training using sets of 1-to-1 orthologs its application to species with very highly diverged genomes will be limited due to the low number of 1-to-1 orthologs. We expect that the method will be applicable within the large groups of eukaryotes, for example, vertebrates, land plants, ecdysozoa.

26) If "the algorithm can be applied to Ribo-seq data, proteomic data, etc." please show evidence for this, by running the method on such data and obtaining relevant results.

Currently there are no such datasets available for plants used in our study (the detailed proteome map is available only for Arabidopsis) so we can not make such direct comparison. We revised this sentence and added the notion that this is our expectation.

27) Similarly, the method would be more convincing if the authors would show application to more than two species.

We agree (and other reviewers also pointed out this issue) and did this. We performed additional analysis by running ICML procedure on two other pairs of species, which have greater evolutionary distance (150-200 m.y.a). The distant species is represented by Zea mays (maize), an agriculturally important plant for which extensive transcriptomic data are available. So we added two more pairs: Arabidopsis - maize and buckwheat - maize. The efficiency of classifier is high for these pairs as well as for Arabidopsis-buckwheat initially sampled in the manuscript.

28) The use of results from a single orthology method as input is a potential major weakness of the approach. The authors should test the sensitivity to different orthology

methods. Moreover, they explain that orthology methods all have shortcomings, but then they use one of them as input, so it isn't clear how this new approach will help.

We tested the applicability of our approach based on alternative orthology inference method (Proteinortho). The results show that the efficiency of classification and its outcome are similar to that inferred from previously used method (Orthofinder). As for the shortcomings of the orthology inference methods, we do not mean that they are unreliable but rather that they do not allow to infer 1-to-1 relationships for most genes in the genomes which have large gene families due to whole genome or segmental duplication. We revised the text adding this information.

29) The training of the machine learning is very limited. On the one hand are orthologs which are assumed to have similar expression (whereas in other parts of the manuscript this is presented as a hypothesis to test). On the other hand are random pairs which are not matched in any way: not by gene length, not by level of expression, not by GC content, etc. It is trivial that with this approach the orthologs will be way more similar than the random pairs.

We actually do not present the assumption that the orthologs have similar expression profiles as a hypothesis; we just note that in some cases (quite rare, 6% according to our estimates) this is not true. The key idea of our algorithm is to find which of the co-orthologs are more functionally similar to each other than the others taking the expression as an indication of function. We thus do not take into account other features. This is, of course, expected and might be called trivial, that the 1-to-1 orthologs are more similar by their expression profiles than random pairs but this is not our result, this is our premise. Using it for training we aim at finding the pairs of co-orthologs which are as similar as 1-to-1 orthologs are and ones which are dissimilar. We find a lot of cases of dissimilar expression patterns within orthogroups - which is not trivial and indicates that despite the general similarity of orthologs (even 1-to-many or many-to-many) by a lot of parameters like length and GC-content their expression profiles are divergent.

30) I was shocked to find that the code on Github is hard coded for the two species studied here, e.g.:

```
my $currAthNam = $athList[$athIndex];

my $currFescNam = $fescList[$fescIndex];
```

That’s our bad, we corrected this.

31) Why are read counts not normalized? This is going to leave a large effect of gene length, since longer genes will have more reads for the same level of expression.

The methods of normalization which account for sequencing depth and length are by definition the multiplication on a constant - this procedure is not expected to change the outcome because we compare not absolute expression values but the the expression profiles which are based on the ratio of read counts in different samples.

32) Why use "an approach similar to cross-validation" rather than cross-validation?

we corrected this

33) How are alternative transcripts of the same gene treated for read counting?

Current implementation of ICML does not include information on alternative splicing; one transcript per gene (the one encoding longest ORF) is considered.

34) How is the method sensitive to the specificity (e.g. tissue-specificity) of expression of genes? Is it better or worse at classifying broadly expressed genes?

We performed the test by selecting genes with broad an narrow expression patterns and analysing the success of classification for these two groups separately. The results showed that genes with broader expression patterns are classified better than ones with narrow pattern. The effect is more pronounced for the comparisons that include maize because transcriptome map of maize includes includes set of samples which are less well matched with samples of Arabidopsis and buckwheat transcriptome maps than these two latter are matched between themselves (in particular maize map is focused on root developement, though contains abode-ground vegetative and reproductive organs as well). This is expected because if a gene is expressed in some narrow specific pattern, it may miss from the transcriptome map is the set of samples is not exhaustive. We included these results in the manuscript.

35) Several references, when checked, do not say what they are cited to say. This is worrying, as I didn't check all references.

- ref 19 isn't a test of method efficiency, but a general public review.

  We agree and indicated that in the text; also we added several references specifically dealing with expression data

- ref 20 used XGBoost for "Natural language processing of text from GEO series" in a paper on crowd-sourcing, not expression data.
- ref 21 doesn't have any reference to XGBoost nor to machine learning as far as I could tell (rapid scan and search for terms in the full text).

  We agree and removed these references and also re-checked other references in order to avoid mis-citing.

- refs 11,28 aren't wrongly cited, but being from 2012 and 2016 cannot be used line 253 to show "growing interest".

  We added more references including more recent ones

https://bmcecolevol.biomedcentral.com/articles/10.1186/s12862-015-0426-x

Кессман! https://www.ncbi.nlm.nih.gov/pmc/articles/PMC6658352/

36) I would like to see a reference for the statement lines 257-258 that plants are more diverse in morphology than animals, and thus matching of samples is more difficult. The Plant Ontology is certainly much simpler than Uberon (bilaterian animals), and my experience runs contrary to the statement of the authors.

This is difficult to get a single reference because there are very little works that directly compare complexity in plants and animals. Our arguments for this is that plants have indeterminate growth and their development is much less constrained and more influenced by the environment (let alone the alternation of halpoid and diploid generations typical for seedless plants). Also, in plants there are many cases of organs with intermediate characteristics (this led to a concept of “dynamic morphology” developed by Rolf Sattler). However we agree that this is a debatable question and that this might not be true for all animals so we modified this sentence.

Minor comments

line 78-79: what justifies the remark about regulatory genes?

We removed this statement.

This does not prevent the influence of random fluctuations, it mitigates it; to control for random factors, you can fix the seed for random generators: "To prevent the influence of random fluctuations on the algorithm output, we performed 100 independent iterations of the training and ES calculations"

The goal of the iterations was specifically to prevent mis-classification of the elements of negative set that have high similarity of expression (this may occur due to the widespread existence of coexpression). We added the despcription of this issue in order to make it clearer.

line 178 "neofunctionalisation" is usually defined between paralogs, not between orthologs.

We fixed this.

line 230: either there broad similarity of function among orthopairs, or "widespread inconsistency", but not both.

We agree, the fraction of such pairs is ~ 6%, so we modified the text accordingly.

Data availability

Have the authors made all data and (if applicable) computational code underlying the findings in their manuscript fully available?The PLOS Data policy requires authors to make all data and code underlying the findings described in their manuscript fully available without restriction, with rare exception (please refer to the Data Availability Statement in the manuscript PDF file). The data and code should be provided as part of the manuscript or its supporting information, or deposited to a public repository. For example, in addition to

summary statistics, the data points behind means, medians and variance measures should be available. If there are restrictions on publicly sharing data or code —e.g. participant privacy or use of data from a third party—those must be specified.

Reviewer #1: **No:** Code not fully available. See comments to authors.

We fixed this

Reviewer #2: **No:** I could not find what are the exact datasets used for this study. Specifically, which expression datasets were used for each species?

We added this information (see Tables S4, S5)

Reviewer #3: Yes
