# Supplementary material for: Interspecific comparison of gene expression profiles using machine learning
Source: PLoS Comput Biol. 2023 Jan 10;19(1):e1010743. doi: 10.1371/journal.pcbi.1010743 (PMC9879537; doi:10.1371/journal.pcbi.1010743)
Supplement: Attachment — Submitted filename: Response to Reviewers.docx [file pcbi.1010743.s018.docx]

Dear Editor,

thank you very much for the feedback on the manuscript. We implemented all the changes requested by the reviewers, including the optimization of our pipeline for working under MacOS and more detailed explanations on the applicability or four approach.

Also, we modified the title of manuscript and the name of a tool, as advised by one of the reviewers during the first round of the review.

Detailed answers to reviewers’ requests are given below.

Reviewer's Responses to Questions

**Comments to the Authors:
Please note here if the review is uploaded as an attachment.**

Reviewer #1: Kasianov et al have done substantial revisions and new experiments:
- They applied a second sequence-based orthology finder (ProteinOrtho).
- The performed the analysis on more pairs of species: Arabidopsis - maize, buckwheat - maize, Arabidopsis - Arabidopsis,
- They applied another ML method (neural networks with custom regularization)
- They introduced a comparison with Euclidean-distance-based method.

The previously missing parts of the code are now included in the repository.

I only have minor issues below that the authors can be trusted to address without another review.

line 68: These processes [...] makes [...] impossible. => These processes [...] make [...] impossible.

- this is corrected

line 158: "input for the algorithm", "First vector is the string". Does the ALGORITHM really use the string rather than the numbers?
I suspect not, although the input to the PROGRAM may be a string representation of numbers, naturally.

indeed, we mean the string representation of numbers and we changed “algorithm” to “program”

line 167: grammar

corrected

line 258: We also implemented ICML pipeline => We also implemented the ICML pipeline

corrected

line 295: we selected the genes with broad an narrow => we selected genes with broad and narrow

corrected

Reviewer #3: In this revision the authors have made many changes and replied to all comments. The manuscript is much improved, and the specific aims of the method are now much clearer.

I still have some comments though.

Major comments:

I was unable to run the method, because the executables from C source did not execute on MacOS, and compilation with gcc gave too many error messages. I could probably fix this, but it is the responsability of the authors to please provide software which functions across platforms.

We modified the program, now it works on MacOS (binary for Mac here: <https://github.com/ArtemKasianov/ICML3/tree/main/bin/MacOS> ).

The authors write several times in the replies to reviewers that the input samples should cover a wide range of conditions, and that they should be from species with sufficient similarity, but this is never clearly defined. The authors should provide specific guidance on the conditions under which their method is expected to work.

We included the paragraph discussing that. The complete range of the variation of body plans however actually depends on the research questions (e.g. it seems quite pointless to compare Arabidopsis and a fruit fly in terms of the expression profiles in their adult body structure, but totally adequate and informative – in circadian cycle, or reaction to stress etc).

Related, please remove the claim page 19 that the method removes the limitation of comparing similar species, since similar body plans are needed, and the plants compared are not more distant than the divergence among mammals.

We can not completely agree with this comment because, though indeed our approach requires the similarity of a general body plan, even within this general body plan there is a lot of variation which makes the direct matching impossible. For example, common buckwheat, one of the objects sampled in our study, has a perianth not differentiated into petals and sepals, while another object, Arabidopsis thaliana has differentiated perianth. The perianth of buckwheat thus does not correspond to either sepals or petals of Arabidopsis thaliana. Also, there are many cases with unclear/mixed homology, e.g. <https://link.springer.com/article/10.1007/s10265-018-01082-7>, Prenner et al. 2008: <https://www.journals.uchicago.edu/doi/abs/10.1086/588071> ). We added several sentences discussing that.

In the text the authors still mention sub and neo-functionalisation several times, whereas their method does not allow to test such outcomes. I suggest removing all such mentions. Similarly, line 66, genes can be retained by not neo or sub-functionalised (e.g. selection for dosage), please remove this.

We removed the mentions of sub and neo-functionalisation in the abstract and modified the sentence in the introduction.

Minor comments:

The authors write several times "model object" where I think they mean "model species".

corrected

line 85 should be "in recent years this approach was replaced by RNA-seq".

corrected

line 251 "the orthologization" should be "the ortholog detection".

corrected

line 280 "detalization" doesn't exist, do you mean "higher detail"?

corrected

line 303, "is" should be "if" at the end of the line.

corrected

**Have the authors made all data and (if applicable) computational code underlying the findings in their manuscript fully available?**
The [PLOS Data policy](https://journals.plos.org/ploscompbiol/s/materials-and-software-sharing) requires authors to make all data and code underlying the findings described in their manuscript fully available without restriction, with rare exception (please refer to the Data Availability Statement in the manuscript PDF file). The data and code should be provided as part of the manuscript or its supporting information, or deposited to a public repository. For example, in addition to summary statistics, the data points behind means, medians and variance measures should be available. If there are restrictions on publicly sharing data or code —e.g. participant privacy or use of data from a third party—those must be specified.

Reviewer #1: Yes

Reviewer #3: Yes

PLOS authors have the option to publish the peer review history of their article ([what does this mean?](https://journals.plos.org/ploscompbiol/s/editorial-and-peer-review-process#loc-peer-review-history)). If published, this will include your full peer review and any attached files.


If you choose “no”, your identity will remain anonymous but your review may still be made public.


**Do you want your identity to be public for this peer review?** For information about this choice, including consent withdrawal, please see our [Privacy Policy](https://www.plos.org/privacy-policy).

Reviewer #1: **Yes:** Mario Stanke

Reviewer #3: No
